# Treatment pathways and rebound-rate of prehospital viral croup attacks—data from a prehospital pediatric physician led emergency service—a prospective observational follow-up study

**DOI:** 10.3389/fped.2025.1544480

**Published:** 2025-05-12

**Authors:** Florian Hey, Victoria Lieftüchter, Martin Olivieri, Sebastian Zimatschek, Florian Hoffmann, Daniel Pfeiffer

**Affiliations:** ^1^ Department of Pediatric Intensive and Emergency Care, Dr. v. Hauner Children’s Hospital, LMU University Hospital, LMU Munich, Munich, Germany; ^2^ Prehospital Pediatric Emergency Service, Munich, Germany

**Keywords:** croup, pediatric emergency medicine, emergency medical services, prehospital/EMS, respiratory diseases

## Abstract

**Introduction:**

Respiratory illnesses, often caused by upper or lower airway obstruction, represent one of the most common pediatric emergencies. Croup syndrome is the most frequent cause of inspiratory stridor. The study aims to record the incidence, current treatment, and further care measures. Additionally feasibility and suitability of future telemedical consultations for pseudo-croup syndrome should be evaluated.

**Methods:**

A prospective observational follow-up study of children aged 0–18 years who were seen by the Munich physician-led prehospital pediatric emergency service from October 15, 2020 to April 30, 2023. The attending emergency physician completed an anonymous questionnaire with treatment information. The child's parents provided a second questionnaire regarding the clinical course and further care in the 12 h following the initial presentation.

**Results:**

A total of 226 patients, 154 (68.1%) with a corresponding parental questionnaire, were analysed. The average age was 3.4 years (range 5 months to 9.5 years), with most patients in the toddler (37.6%; *n* = 85) and early childhood (45.1%, *n* = 102) age brackets. 8.4% (*n* = 19) of patients had a, most frequently respiratory (52.6%, *n* = 10), chronic precondition. The average Westley Score in our cohort was 4.1. Every year increase in age reduces the average Westley score by 21.2% (*p* = 0.034). Acute therapy consists of steroids rectally (98.2%, *n* = 222), adrenaline (85.8%, *n* = 194) and cold/fresh air (78.8%, *n* = 178) inhalations. 39.8% (*n* = 90) of patients were transported to the hospital, and a physician accompanied a third (35.6%, *n* = 32). The strongest predictor for the necessity of physician-accompanied transport was prolonged adrenaline inhalations (OR: 11.25). Nearly ¾ of patients (70.2%, *n* = 47) were discharged from the emergency department. Of all admitted patients (*n* = 20), 10% (*n* = 2) needed intensive care. Out of all patients (*n* = 226), in 7% (*n* = 10) of cases with parental information on reoccurrence, a reoccurrence of the croup attack within 12 h was reported.

**Conclusion(s):**

Croup syndrome attacks have a low risk of hospitalisation and rebound. In light of increasingly limited healthcare resources, this study identifies several significant influencing variables for the treatment pathways and proposes a potential treatment algorithm. No patient needed invasive treatments, rendering croup attacks in children a possible target for telemedical consultations with no necessity for on-site physician presence.

## Introduction

Respiratory and airway emergencies account for approximately 19% of prehospital pediatric emergencies requiring an emergency physician. Roughly 46% are due to Croup syndrome, also known as viral laryngotracheitis. Croup is the primary cause of acute upper airway obstruction in children, affecting around 3% of young children, usually between 6 months and 3 years, and accounting for about 7% of hospitalisations^1^ (1–3).

Typical symptoms of a viral croup syndrome are sudden onset (often at night), inspiratory stridor, “barky” cough, hoarseness, dyspnea, upper respiratory tract infection and mostly slightly restricted clinical condition. The highest incidence of croup is seen in the autumn and early winter months between September and December. Life-threatening situations can occur quickly due to physiologically narrower airways in children, higher oxygen requirements, and significantly lower functional residual capacity with lower reserves. Mortality, however, is very low at 0.5%. The therapy of inspiratory stridor, with its three main pillars—inhalation of cold/fresh air, adrenaline and systemic steroids — aims to decongest the airways as quickly as possible (see Footnote 1) (2–5).

Health policy and economic aspects, among other things, lead to staffing shortages, decreased numbers of available hospital beds and more frequent emergency department overcrowding, particularly in the infection period and winter months. Therefore, this study aims to explore the characteristics that determine treatment pathways and the frequency of rebounds. Furthermore, this study aims to analyse the feasibility and suitability of future telemedical consultations to facilitate a more efficient and geographically broader resource use. The current literature is sparse and no relevant studies in recent literature cover similar topics in a prehospital setting.

## Material and methods

Prospective cross-sectional observational study of children aged 0–18 years who were seen by the Munich prehospital physician-led pediatric emergency service from October 15, 2020 to April 30, 2023. The pediatric emergency physician service is a 24/7 specialised prehospital ambulance unit in Munich, Germany, deployed by the Munich Emergency Services Dispatch Center by car or helicopter to every child up to 12 years of age in life-threatening conditions. Children with acute but not potentially life-threatening conditions are not regularly seen by the pediatric emergency physician. Paediatricians, pediatric surgeons or anesthesiologists with extensive pediatric intensive care experience and a specialised paramedic staff are equipped with specialised pediatric equipment. After the patient had been medically cared for, the attending emergency physician completed an anonymous questionnaire that included age, severity of croup symptoms using the Westley score (Table 1), risk factors and pre-existing conditions, transport modality, and treatment information. The parents filled out the second part of the questionnaire regarding further care, ED treatment, and reoccurrence information. Both questionnaires can be found in the online appendix—see Supplementary Figures S10, S11. They were sent back anonymously to the study centre in a pre-paid envelope.

**Table 1:** Westley score (1).

Westley Score	0	1	2	3	4	5
Inspiratory stridor	None	When agitated	At rest	–	–	–
Retractions	None	Mild	Moderate	Severe	–	–
Air entry	Normal	Decreased	Markedly decreased	–	–	–
Cyanosis	None	–	–	–	When crying	At rest
Level of consciousness	Alert	–	–	–	–	Disoriented

Data was entered into Microsoft Excel and analysed in IBM SPSS using basic descriptive statistics, percentages and binary logistic regressions with stepwise backward selection to identify the most influential factors and account for confounding. Furthermore the Kruskal–Wallis-Test was used to test for significance in groups.

Out of the 249 questionnaires handed out, 17 did not arrive in the study centre. For one patient, only the parental questionnaire was on file. One case had no correct age, and another was missing information on transport modality. Three cases in which the patient drove to the hospital with parents were excluded from the study. Thus, 23 patients were excluded from the final study population. In the final study population, 11 cases (4.8%) had incomplete information on the Westley score.

The Westley score is an internationally recognised classification for scoring the severity of croup attacks. See Table 1. A Westley score less than or equal to 2 indicates mild croup. A Westley score between 3 and 5 indicates moderate croup. A Westley score between 6 and 11 indicates severe croup and a score greater than 12 indicates impending respiratory failure (6).

The patient's age was grouped according to the modified Munich Age Classification System (mMACS), in Neonates (≤28 days), Infants (≥29 days–<1 year), Toddlers (≥1 year–<3 years), Early Childhood (≥3 years–<6 years), Late Childhood (≥6 years–<12 years) and Adolescents (≥12 years–<18 years) (7).

## Results

A total of 226 patients with emergency physician questionnaires were included in the study. Of those, 154 (68.1%) had a corresponding parental questionnaire. The average age was 3.4 years (range 0.4–9.5 years). According to the modified Munich Age Classification System (mMACS), most patients are categorised in the Early Childhood or Toddler (45.1%, *n* = 102 and 37.6%, *n* = 85) age bracket. Late Childhood and Infants accounted for 9.7% (*n* = 22) and 7.5% (*n* = 17) of patients, respectively. 8.4% (*n* = 19) of patients had a preexisting underlying disease, most frequently respiratory (52.6%, *n* = 10) in nature (7).

The average Westley Score in our cohort was 4.1. Four patients had a Westley Score of 0, potentially indicating that a croup exacerbation might not have been the correct diagnosis. Age is negatively correlated with the Westley score. A one-year increase in age reduces the average Westley score by 21.2% (*p* = 0.034). Comorbidities are not significantly correlated with the Westley score (*p* = 0.940).

Almost all patients (98.2%, *n* = 222) seen by the emergency paediatrician in a prehospital setting with croup-like symptoms received Prednisolon (steroids) rectally. 85.8% (*n* = 194) received adrenaline inhalations. Cold/fresh air was applied in 78.8% (*n* = 178) cases. In 9 cases, additional treatments were used, this included other inhalative agents, like Salbutamol (5×), ipratropium bromid (1×), inhalative steroids (1×), NaCl 0.9% (1×) and NaCl 3% (1×), as well as Ibuprofen (2×) and acetaminophen (1×). Patients with higher scores in the subsection retractions (OR: 2.92) and cyanosis (OR: 1.37) had an increased need for prolonged adrenaline inhalations. We observed a trend that patients who needed additional oxygen also needed prolonged adrenaline inhalations (OR: 7.33). However, this was not significant (*p* = 0.065).

In 8 cases (3.5%), additional oxygen application beyond the short oxygen application needed for inhalation was necessary. The average Westley score of these patients was 8.63, with an average age of 5 years (range 2.5–8.5 years). Prolonged adrenaline inhalations (OR: 31.98), higher scores in the subsection cyanosis (OR: 3.08) and higher age (OR: 2.67) were significantly positively associated with a need for additional oxygen. Cold/fresh air application (OR: 0.12) is significantly associated with decreased oxygen need. We observed four patients treated only with cold air (average Westley Score of 3.5). All of them remained at home, and none of them suffered a reoccurrence.

**Figure 1:**
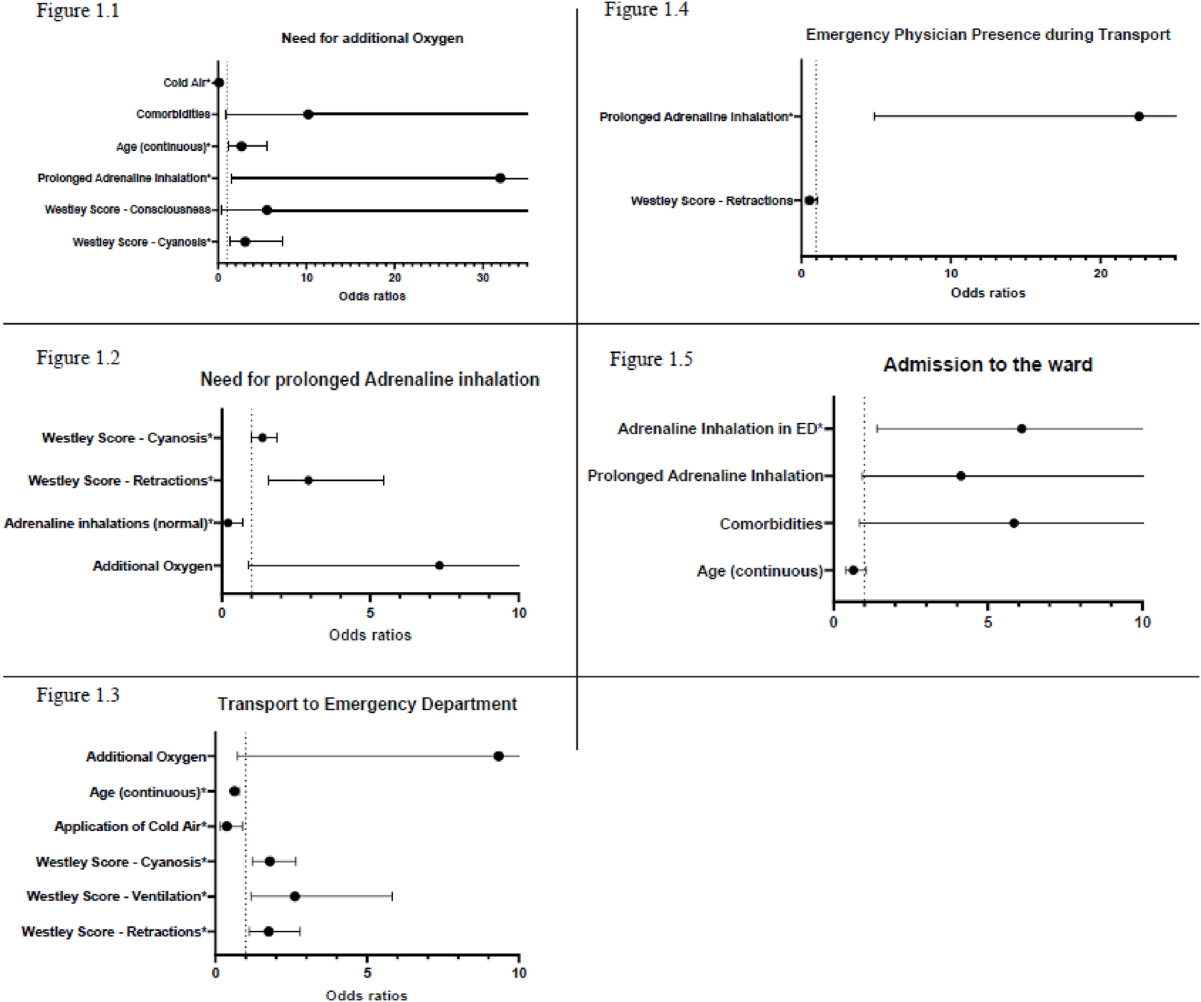
Binary logistic regressions with stepwise backwards selection.

39.8% (*n* = 90) of all patients seen by the service were transported to a hospital, of which the pediatric emergency physician accompanied 35.6% (*n* = 32). See Table 2 for further details. Patients of higher age (OR: 0.63) and those who inhaled cold air (OR: 0.382) were significantly less frequently transported to the emergency department. In contrast, those with higher scores in the subsection of the Westley Score retractions (OR: 1.76), cyanosis (OR: 1.80) and ventilation (OR: 2.62) were significantly more frequently transported to an ED (OR: 1.76). We observed a trend that the need for additional oxygen (OR: 9.337, *p* = 0.085) also increases the likelihood of being transported to an ED (see Figure 1).

**Table 2:** Characteristics of patient transport indication and modality.

Influencing factors for patient care pathway	The patient stayed at home *n* = 136	Transport to hospital without emergency doctor *N* = 58	Transport to hospital with emergency doctor *N* = 32
Average Westley Score*^,^**	3.1	5.0	6.8
% showing cyanosis*^,^***	2.2	15.3	46.9
% needing oxygen*^,^****	0.7	3.4	15.6

* Kruskal–Wallis-Test *p* = 0.001.

** Significant differences in the comparison of Transport to hospital without an emergency doctor compared to Staying at home (*p* = 0.000, medium effect size) and Transport to a hospital with an emergency doctor compared to staying at home (*p* = 0.000, medium effect size). There is no significant difference between categories of transport with and without an emergency doctor (*p* = 1.000).

*** Significant differences in all subcategories. Staying at home – Transport without the emergency doctor (*p* = 0.028, weak effect size), Staying at home—Transport with the emergency doctor (*p* = 0.000, strong effect size), Transport with the emergency doctor—Transport without the emergency doctor (*p* = 0.000, medium effect size).

**** Staying at home—Transport with emergency doctor (*p* = 1.000). Staying at home—Transport with emergency doctor (*p* = 0.000, medium effect size). Transport with—Transport without emergency doctor (*p* = 0.008, medium effect size).

**Figure 2:**
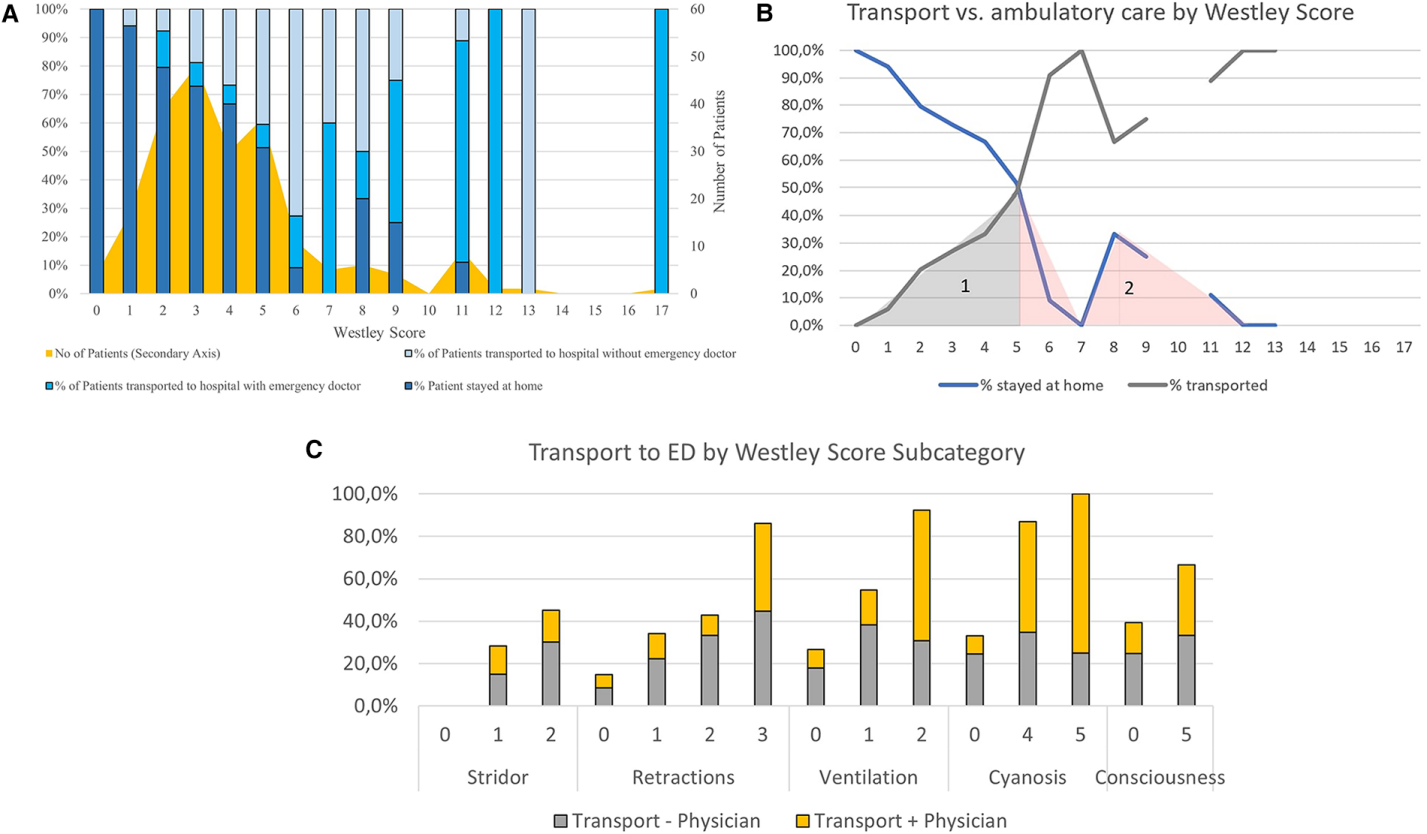
**(A)** – Patient transport pathway according to Westley Score. **(B)** – Transport vs. ambulatory care by Westley Score. **(C)** – Transport to ED by Westley Score subcategory.

Figures 2A–C show our study cohort's patients' care pathways. Figure 2B suggests a cut-off value of 5 on the Westley Score to differentiate between ambulatory care and transport to a hospital. In our cohort, a rule to transport >5 on the overall Westley Score would mean that 50 patients who were transported to a hospital, would not have been transported to a hospital (Area 1). In comparison, four patients who stayed home would be transported to a hospital.

Prolonged adrenaline inhalations were significantly more often associated with emergency physician presence during transport (OR: 11.250). An increase in the Westley score leads to a significantly higher transport rate to a hospital (*p* = 0.001, OR: 1.7). See Table 2.

Out of all the patients transported to a hospital (and with corresponding parental questionnaire), the majority (70.2%, *n* = 47) were discharged from the emergency department after an observational period. Only 29.9% of the patients transported to an emergency department (*n* = 20, 0.9% of all patients seen by the prehospital paediatrician) were admitted. Of those, 10% (*n* = 2) were admitted to an ICU. The necessity for adrenaline inhalations in the Emergency Department significantly increases the chance of admission to the ward (OR: 6.1, *p* = 0.016). There was a trend that prolonged adrenaline inhalations prehospitally (OR: 4.13, *p* = 0.062) and comorbidities (OR: 5.846, *p* = 0.074) also increased the likelihood of being admitted, while higher age decreased the likelihood (OR: 0.64, *p* = 0.084). In a separate binary univariate logistic regression, there was a trend that an increase in the overall Westley Score led to an increase in admissions to the ward, although not significantly. (*p* = 0.077, OR: 1.2) Out of all patients with a Westley Score higher than seven, 81.8% were transported to the emergency department and 43.8% were admitted to the ward, compared toa transport rate of 34% and an admittance rate of 25.5% for those with a Westley Score of smaller or equal to seven.

In 7% (*n* = 10) of all cases seen by the prehospital physician, with parental information on reoccurrence, there was another croup attack within 12 h of initial contact. The average Westley Score of patients suffering a reoccurrence was 3.9 (range 2–8). 9 out of 10 patients were in the Toddler or Early childhood category. All patients with croup reoccurrences received steroids and adrenaline inhalations. 2 patients needed prolonged adrenaline inhalations, and one patient needed additional oxygen. Eight of the ten patients stayed home; the remaining two were discharged from the emergency department after examination. No patient that was admitted had a reoccurrence within 12 h. Due to the low total number of reoccurrences within 12 h captured in our data, no reasonable statistical inference could be drawn.

## Discussion

Our data confirm good adherence to established treatment protocols for croup exacerbations. Steroids, inhalative adrenaline, and cold air were frequently applied. Due to the unavailability of oral steroids, no use of oral dexamethasone was reported in our study. In contrast, other studies observed an application rate of around 10.6% of children arriving in the ED with a croup diagnosis. The application of oral rather than rectal steroids might be associated with less stress and agitation, further contributing to the calm management of croup attacks (3, 8).

The simple and quick intervention of applying cold air significantly and strongly decreases the need for additional oxygen and transport to an ED. Cold air is a simple yet powerful measure to reduce oxygen needs and reduce clinical symptoms. Measuring body temperature and heat preservation in small children is necessary to avoid hypothermia. On the other hand, cold air could be recommended by EMS personnel through telemedicine. However, it could also be true that when a child is more severely ill, e.g., needs oxygen, medical personnel less frequently consider applying cold air. Additionally, to cold air, glucocorticoids, even in low doses like 0.15 mg/kg, have been shown to reduce the symptoms of a croup attack after approximately two hours. There are multiple treatment algorithms suggested in the literature but there is no clear treatment algorithm adapted on the pediatric emergency physician car, the physician decides individually on a case-by-case basis, which is why multiple pathways are analysed in our study (1, 9–11).

Yang et al. (12) suggested a Westley Score of 1–2 for treatment at home and a Westley Score of >5 as an indication for hospitalisation. With an average score of 3.1 of our patients staying at home, our data cannot confirm these findings, suggesting that a much larger cohort of patients can be treated in an outpatient setting. This would significantly contribute to reducing the strain on healthcare resources. Emphasis must, however, be placed on the fact that an emergency pediatrician saw the patients in their home. This could not be uncritically extended to other emergency care systems as longer distances to reach a hospital in case of deterioration may impact patient safety. The patients in our cohort who were transported to an ED had an average Westley Score of 5.6, which is comparable to the findings of Yang et al. (12). Patients admitted to the ward had an average Westley Score of 7, suggesting that the Westley Score is an excellent instrument to differentiate between treatment pathways (12).

In our study, most patients had no reoccurrence, supporting the decision pathway for an outpatient treatment without hospitalisation. Out of all patients transported to a hospital, only one-third were admitted to a ward, of which none reported a reoccurrence within 12 h. This observation is also highly relevant to inform the decision to transport a child to the hospital.

Interestingly, higher age led to a more frequent need for additional oxygen (OR: 2.67), while simultaneously being significantly less frequently transported (OR: 0.63). This contrary trend might be due to the low number of administrations of additional oxygen in the observed study cohort (*n* = 8). The average age of these eight patients receiving additional oxygen (above inhalations) was 5 years, with an average Westley Score of 8.6. Seven out of those eight patients were transported to a hospital. For those with corresponding parental questionnaires, 33.3% (2 out of 6) were admitted to the ward. Combining these results with perceived experiences, older children might be less severely impaired by croup, most likely due to wider airways.

No patient in our study cohort needed invasive procedures (for example, intravenous access or airway management). Therefore, croup syndrome is an illness that paramedics can potentially treat with reasonable safety with the telemedical assistance of a pediatric emergency physician. The literature describes an hospital admission rate of less than 5% (from 1.5% to 31% of children seen in an outpatient clinic). Only 1%–3% of admitted patients require intubation. <0.5% of all intubated patients die due to respiratory failure (approximately 1 in every 30.000 cases). Similar recurrence rates of 5% were described in the literatue (13–16).

**Figure 3:**
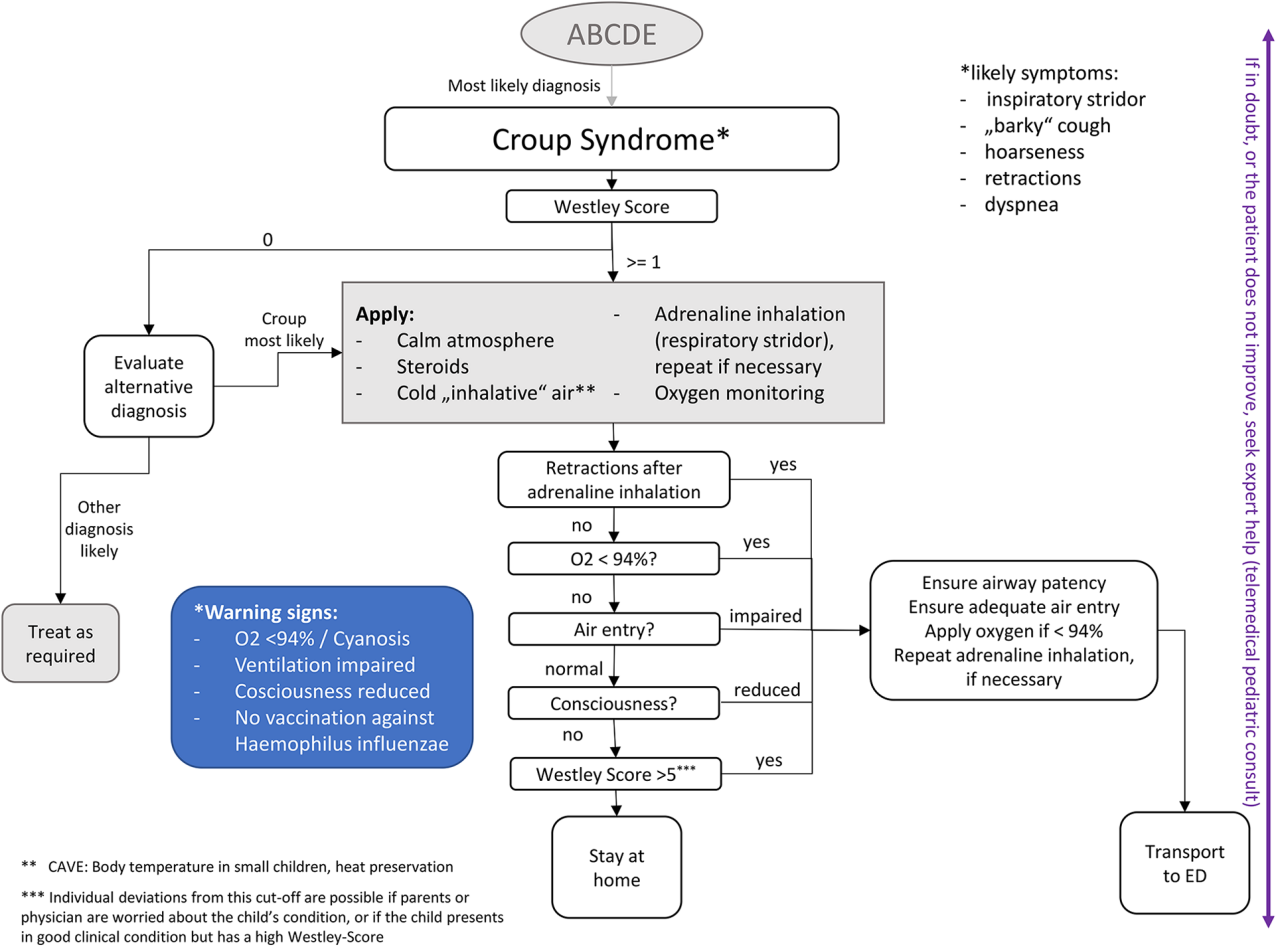
Potential treatment algorithm.

Based on our data, we derived the following treatment algorithm for croup syndrome in a prehospital setting (see Figure 3). In general, we propose a cut-off value of >5 on the Westley scale to initiate transport to a hospital. The most frequent indications for transport to hospital were cyanosis and need for oxygen, both are markers for respiratory insufficiency. According to our regression outputs, all children who need more than one inhalation of adrenaline should be transported to the emergency department. Younger children and those with comorbidities might also be more likely to be transported to a hospital. This algorithm is intended to guide the creation of specific algorithms adapted to the unique requirements of each prehospital service environment. Our results show that 50% fewer patients would have been transported to a hospital using our algorithm, relieving the pressure on ambulance services and emergency departments. Further evaluation of the proposed treatment algorithm is necessary.

Zoorob et al. (14) propose a more stepwise approach tailored to potentially less severe cases seen by general practitioners. Our proposed algorithm was developed with data from a specialised prehospital emergency service that sees only the sickest of patients. Both algorithms, therefore, have their justification and use cases (14).

Cold air is an essential and powerful intervention to derive concrete, practical implications from our data. Retractions, impaired ventilation or consciousness and cyanosis are important warning signs indicating a more severe croup attack. If no sufficient improvement is noted (e.g., remaining stridor, retractions, supplemental oxygen) after the application of steroids, cold air and adrenaline inhalation, a physician should evaluate whether transport to a hospital is necessary.

## Limitations

This study has multiple limitations. The parental questionnaire is not available for all patients presenting with croup-like symptoms seen by the physician-led pediatric emergency service, introducing a potential underreporting bias. Some printed questionnaires did not arrive at the study centre. It remains unclear whether they were handed out to patients or were lost at some other point before being handed out, also introducing a potential underreporting bias. The assessment of a croup rebound by non-medically trained parents might not be completely accurate. Differences in the experience of the emergency physician introduce subjectivity and variability in the chosen treatment paths. While experienced physicians might be more prone to leave a child at home, relatively more inexperienced physicians might start inhalations sooner. This is also true for reporting emergency department procedures (wait times and treatments) by non-medically trained parents. The pediatric emergency physician car did not carry dexamethasone orally during the study period. To apply steroids, the physician has to either resort to rectal or intravenous application methods. There was no clear differentiation between “adrenaline inhalation” and “continuous/prolonged adrenaline inhalation” provided on the questionnaire and no clear definition of inhalation dosing and amount, resulting in a potentially blurry distinction between the two categories. However, we believe that prolonged inhalation was reported when the first adrenaline inhalation had to be refilled, which could be at different times of the treatment due to varying initial doses. Dosing information for adrenaline was not included in the study questionnaire due to the widespread use of a pure formulation for inhalation. Due to the monocentric nature of the dataset and the highly selective inclusion criteria of patients seen by one singular, highly specialised pediatric ambulance unit, some conclusions might not apply to other regions or the broader population of croup patients seen by regular ambulance crews. It is worth mentioning that most croup patients might not see an ambulance crew at all. Due to the questionnaires being handed out on the scene, croup attacks that do not require medical attention or the attendance of a specialised emergency paediatrician are not included in the study. Only those croup attacks reported were included in the analysis, which introduced an extensive underreporting bias, especially for minor croup events. We have no information on whether the reoccurrence of the croup attack led to a need for interventions or hospitalisation. The study period and results were affected by the COVID-19 pandemic, during which the incidence of croup attacks attended by the emergency physician was strongly decreased.

## Conclusion

Croup attacks have a low rate of necessary hospitalisations and a very low rebound rate within 12 h. This study identifies several significant influencing variables for clinical treatment pathways and proposes a potential treatment algorithm based on the Westley Score. A higher Westley score is associated with an increased risk of prolonged adrenaline inhalations, emergency physician presence on transport and admission. Medical expertise is necessary to identify patients at risk for deterioration and decompensation. Cold/fresh air, steroids and adrenalin inhalation are the pillars of therapy. No patient needed invasive treatments, rendering croup attacks in children a possible target for telemedical consultations with no necessity for on-site physician presence, however, further studies are necessary to investigate the feasibility.

## Data Availability

The raw data supporting the conclusions of this article will be made available by the authors, without undue reservation.

## Footnotes

^1^
Deployment data from the prehospital pediatric emergency physician service in Munich (2017–2020).
