# Supplementary material for: Treatment pathways and rebound-rate of prehospital viral croup attacks—data from a prehospital pediatric physician led emergency service—a prospective observational follow-up study
Source: Front Pediatr. 2025 May 12;13:1544480. doi: 10.3389/fped.2025.1544480 (PMC12104051; doi:10.3389/fped.2025.1544480)
Supplement: Supplementary file 1 [file Datasheet1.pdf]

Figure 11 - Questionnaire Parents

Patient's age:	____________ years _____________ months
Further interventions	after initial treatment by the emergency services wen:<br>stayed at home<br>were taken to hospital by ambulance
What other measures were carried out in the **hospital** (excluding therapies by the emergency services)	cold air<br>rectodelt (rectal steroids) supp.<br>dexamethason orally<br>adrenaline inhalation<br>multiple adrenaline inhalations/constant adrenaline inhalation<br>others ________________________
Further treatment	discharge home after examination in clinic<br>monitoring for up to 2 hours in the emergency department<br>monitoring for 2 to 4 hours in the emergency department<br>monitoring for > 4 hours in the emergency department<br>in-patient admission to the normal ward<br>in-patient admission to the intensive care unit
Course of disease	No new croup attack in the first 12 hours<br>Croup occurred again in the first 12 hours
Is there anything else you would like to tell us?	
