# Supplementary material for: Treatment pathways and rebound-rate of prehospital viral croup attacks—data from a prehospital pediatric physician led emergency service—a prospective observational follow-up study
Source: Front Pediatr. 2025 May 12;13:1544480. doi: 10.3389/fped.2025.1544480 (PMC12104051; doi:10.3389/fped.2025.1544480)
Supplement: Supplementary file 2 [file Datasheet2.pdf]

Figure 10 - Questionnaire Emergency Physician

<table>
<tr><td>Patient‘s age:</td><td colspan="2">____________ years _____________ months</td></tr>
<tr><td>Comorbidities</td><td colspan="2">☐ none
☐ respiratory comorbidities
☐ cardiac comorbidities
☐ difficult upper airway
☐ other comorbidities</td></tr>
<tr><td rowspan="5">Severity
(Westley Score)</td><td>Stridor</td><td>☐ none
☐ with agitation
☐ at rest</td></tr>
<tr><td>Retractions</td><td>☐ none
☐ mild
☐ moderate
☐ severe</td></tr>
<tr><td>Air entry</td><td>☐ normal
☐ decreased
☐ markedly decreased</td></tr>
<tr><td>Cyanosis</td><td>☐ none
☐ with agitation
☐ at rest</td></tr>
<tr><td>Level of consciousness</td><td>☐ normal
☐ disoriented</td></tr>
<tr><td>Therapy</td><td colspan="2">☐ cold air
☐ rectodelt (rectal steroids) supp.
☐ adrenaline inhalation
☐ multiple adrenaline inhalations/constant adrenaline inhalation
☐ other _______________________</td></tr>
<tr><td>Additional interventions</td><td colspan="2">☐ oxygen therapy (excl. inhalation therapy)
☐ bag-mask ventilation
☐ intubation
☐ other ______________________</td></tr>
<tr><td>Further treatment</td><td colspan="2">☐ patient stays at home
☐ parents drive patient to the emergency department
☐ transport with ambulance without emergency physician
☐ transport with ambulance and emergency physician</td></tr>
<tr><td>Comments</td><td colspan="2"></td></tr>
</table>
